# How to improve the prognosis of high‐risk elderly gastric cancer patients

**DOI:** 10.1002/ags3.12627

**Published:** 2022-10-03

**Authors:** Hiroya Takeuchi, Eisuke Booka

**Affiliations:** ^1^ Department of Surgery Hamamatsu University School of Medicine Shizuoka Japan

Currently, Japan is facing various challenges in medical care, such as an aging population and an increase in medical expenses. Among the critical challenges in surgical treatment in Japan, one is surgery for elderly patients with cancer, and the other is the high cost of robotic surgery, the cutting‐edge medical care. Treatment strategy for elderly patients with cancer is extremely challenging owing to the patient's organ dysfunction and life expectancy. Currently, at several Japanese institutions, treatment strategies are not determined solely based on age; elderly patients with cancer are likely to be treated in the same manner as younger patients with cancer if their organ function is preserved and their performance status (PS) is satisfactory. However, with age, the organ functions of patients with cancer naturally decline, making multidisciplinary treatment challenging. Patients with elderly cancer often cannot receive neoadjuvant and adjuvant chemotherapy; surgery has a greater impact on the prognosis of elderly patients with cancer than that in younger patients.

In this issue of the *Annals of Gastroenterological Surgery*, Iida et al reported the risk factors for non‐gastric‐cancer‐related death after gastrectomy in elderly patients.^1^ The risk factors for non‐gastric‐cancer‐related death and overall survival of patients aged ≥75 years who undergo radical gastrectomy are male gender, low skeletal muscle mass index (SMI), and high neutrophil‐to‐lymphocyte ratio (NLR). According to the Report of the National Clinical Database 2011‐2019, 16.8% of the patients who underwent a distal gastrectomy in 2011 were aged 75‐80 years, and 18.5% were aged ≥80 years compared to 19.3% aged 75‐80 years and 24.3% aged ≥80 years in 2019.^2^ Moreover, 18.0% of the patients who underwent a total gastrectomy in 2011 were aged 75‐80 years, and 15.0% were aged ≥80 years compared to 21.5% of patients who were aged 75‐80 years and 20.6% were aged ≥80 years in 2019.^2^ Distal and total gastrectomies for elderly patients are increasing annually; however, attention must be given to non‐gastric‐cancer‐related deaths in gastrectomy for elderly patients. When performing gastrectomy for male patients with gastric cancer with low SMI or high NLR, efforts are needed to prevent death from other diseases, such as reducing postoperative complications, performing minimally invasive surgery, and preventing PS worsening.

This issue also reports on modifications in the number of robotic gastrointestinal surgeries owing to insurance coverage.^3^ Robotic gastrointestinal surgery is reimbursed by the National Health Insurance system since April 2018 in Japan. The number of patients who underwent robotic surgeries each month in 2018 remained stable in the low 100 s between January and March, while it nearly doubled in April (n = 221) and quadrupled in October (n = 432). Robotic total, distal, and proximal gastrectomies illustrate the same upward trend. Insurance coverage has increased robotic gastrectomy (RG) dramatically. Suda et al reported safe implementation of insurance‐covered RG nationwide.^4^ RG would become popular with insurance approval and is anticipated to become even more popular in the future. Japan's universal health insurance system has various challenges, such as an increase in the burden of medical expenses; however, it has played a major role in the RG spread. Similar to laparoscopic surgery, any technique evolves through popularization. The technique of RG is anticipated to progress more in the future with its popularization. The superiority of RG should be demonstrated compared to laparoscopic gastrectomy as evidence derived from randomized controlled trials or big data.

Elderly patients with gastric cancer often undergo only gastrectomy without neoadjuvant and adjuvant chemotherapy; gastrectomy alone determines the patient's prognosis. It is anticipated that the introduction of minimally invasive surgery by RG may not only improve the curability of gastric cancer but also prevent death from other diseases and improve the prognosis of high‐risk elderly patients with gastric cancer, e.g. in men with low SMI and high NLR.

## DISCLOSURE

Conflict of Interest: Hiroya Takeuchi received lecture fees from Johnson&Johnson, Medtronic, Ono Pharmaceutical, Taiho Pharmaceutical, and Daiichi Sankyo, and received research expenses from Taiho Pharmaceutical, Chugai Pharmaceutical, and Otsuka Pharmaceutical Factory.
